# Primary hypertrophic osteoarthropathy related gastrointestinal complication has distinctive clinical and pathological characteristics: two cases report and review of the literature

**DOI:** 10.1186/s13023-019-1264-5

**Published:** 2019-12-26

**Authors:** Qiang Wang, Ying-he Li, Guo-le Lin, Yue Li, Wei-xun Zhou, Jia-ming Qian, Wei-bo Xia, Dong Wu

**Affiliations:** 1 Department of Gastroenterology, Peking Union Medical College Hospital, Chinese Academy of Medical Sciences, Beijing, China; 2 Department of General Surgery, Peking Union Medical College Hospital, Chinese Academy of Medical Sciences, Beijing, China; 3 Department of Pathology, Peking Union Medical College Hospital, Chinese Academy of Medical Sciences, Beijing, China; 4 Department of Endocrinology, Peking Union Medical College Hospital, Chinese Academy of Medical Sciences, Beijing, China

**Keywords:** Primary hypertrophic osteoarthropathy, Gastrointestinal, Pathology, Review, CEAS, CMUSE, Crohn’s disease

## Abstract

**Background:**

Primary hypertrophic osteoarthropathy (PHO) is a rare disease related to *HPGD* and *SLCO2A1* gene mutation. Gastrointestinal involvement of PHO is even rarer with unknown pathogenesis. Clinical features of GI complication in PHO mimics other auto-immune based bowel entities, such as inflammatory bowel diseases and cryptogenic multifocal ulcerous stenosing enteritis (CMUSE). We aimed to analyze the clinical, genetic, radiological and pathological features of Chinese patients with PHO and determine the difference between PHO patients presenting with and without GI involvement.

**Methods:**

We reported two PHO cases with gastrointestinal involvement and reviewed all the studies of PHO in Chinese population published from January 1, 2000, to April 30, 2018. Clinical and genetic presentations of PHO in Chinese patients were analyzed. We compared the characteristics of those patients with gastrointestinal involvement against those without.

**Results:**

The two patients were both males with complete-form PHO for more than 10 years. GI related symptoms included diarrhea, chronic gastrointestinal hemorrhage, incomplete intestinal obstruction, anemia, and edema, which were unresponsive to etoricoxib treatment. Radiological examinations revealed segmental intestinal stenosis and thickened intestinal wall. Endoscopic findings included multiple ulcers and mucosal inflammation. Both patients had mutations of *SLCO2A1* according to sequence analysis. The surgical pathology revealed chronic inflammation involving the intestinal mucosa and submucosa, similar to histological changes in CMUSE. According to the systemic review of 158 Chinese patients with PHO, 17.2% had gastrointestinal involvement, including peptic ulcer, gastric polyps, hypertrophic gastritis, and segmental intestinal stenosis. Patients with gastrointestinal involvement were more likely to have anemia (40.0% vs. 4.5%, *P* < 0.001), hypoalbuminemia (16.7% vs. 0.9%, *P* = 0.003), and myelofibrosis (19.0% vs. 0.9%, *P* = 0.002) than those without. Most patients with gastrointestinal complication had *SLCO2A1* mutation (86.7%, 13 /15).

**Conclusions:**

Digestive tract involvement is uncommon in patients with PHO and often presents with anemia, and hypoalbuminemia resulted from intestinal inflammation. The intestinal pathologic characteristics are distinct from Crohn’s disease but similar to CMUSE. Mutations in *SLCO2A1* might be the pathogenic cause of GI involvement of PHO. NSAIDs may not be effective for PHO patients with gastrointestinal complications.

## Background

Hypertrophic osteoarthropathy (HO), or pachydermoperiostosis, is a disorder characterized as abnormal growth of skin and bones. It is classified as Primary HO (PHO) and secondary HO according to etiology, with lung cancer being the most common cause of the latter. PHO, which only accounts for 5% of all the HO patients, is a rare genetic disease [1, 2]. In recent years, a body of evidence has shown that mutations of *HPGD* and *SLCO2A1* gene are related with PHO. Both genes encode proteins involving regulation of pro-inflammatory mediators such as prostaglandin. Mutated *HPGD* and *SLCO2A1* genes inactivate prostaglandin transport and degradation, resulting in uncontrolled local accumulation of prostaglandin, especially prostaglandin E2, which is the crucial factor in the pathogenesis of PHO [3, 4].

Clinical features of PHO include digital clubbing, periostosis and pachydermia, with various complications including arthritis, dermatitis, myelofibrosis, and gastrointestinal (GI) abnormalities. It is noteworthy that GI involvement in PHO can mimic other entities including chronic gastritis, peptic ulcer, Crohn’s disease, cryptogenic multifocal ulcerous stenosing enteritis (CMUSE), and chronic enteropathy associated with *SLCO2A1* gene (CEAS). When GI features are the reporting or dominating symptoms in PHO patients, especially when GI features presenting as the initial symptom of PHO, the differential diagnosis may be quite challenging [2]. As *SLCO2A1* is a causal gene for both CEAS and PHO, some of the CEAS patients also have features of PHO, which makes the issue further complicated [5]. Treatments for inflammatory bowel disease, including 5-aminosalicylic acid, corticosteroids, and immunosuppressives are often ineffective for these patients, who frequently require surgery. Thus, timely recognition and definite diagnosis of PHO patients with GI involvement is essential for selection of appropriate therapies [4, 6].

Watery diarrhea has been reported in six Chinese families with PHO [6]. However, information about the clinical and pathological features of GI lesions in PHO remains scarce, much less the pathogenetic mechanism. It seems that PHO patients who have GI complications are usually more severe and more difficult to treat than those who have not, so we aim to examine the difference between PHO patients with and without GI involvement. Here we present two PHO patients with GI involvement as their dominant clinical pattern, who underwent small intestine resections due to severe intestinal hemorrhage and stenosis. The distinguishing radiological, endoscopic and pathological features of GI abnormalities in PHO were presented and analyzed. We also reviewed 158 patients with PHO reported in China in the past 18 years and analyzed their symptoms and complications.

## Methods

### Cases report

Two PHO cases with gastrointestinal involvement admitted to Peking Union Medical College Hospital (PUMCH) at Beijing; China were presented. Both patients received genetic test and surgery with a follow-up period for more than 4 years. The Ethics Committee of the hospital approved the use of the clinical data and genetic test results of the two patients. A consensus had been obtained from both patients to use their pictures, notes and lab investigations for publication on the condition that their personal information was kept confidential.

### Literature search

We conducted a literature search for primary hypertrophic osteoarthropathy or pachydermoperiostosis on PUBMED, EMBASE and Cochrane Library published by Chinese authors and the China National Knowledge Infrastructure (www.cnki.net) database from January 1, 2000, to April 30, 2018. We also checked the reference lists of the studies included and other systematic reviews to identify additional studies.

### Inclusion criteria and data extraction

We included all case reports and original articles for PHO in Chinese patients, which comprehensively described the characteristics of the disease onset and, with or without the information about treatment and prognosis. The titles and abstracts of all the references identified were reviewed independently by two of the authors (WQ, LYH). The full text of the articles considered potentially relevant was then screened and checked for eligibility. Any disagreements about article inclusion were resolved at this stage. We recorded the clinical characteristics, genetic test results, diagnosis, and treatment. Undescribed clinical manifestations were considered absent. We checked the accuracy of data extraction, and any inconsistencies were discussed and resolved.

### Statistical analyses

The Statistical Package for Social Sciences (SPSS), version 13.0 (SPSS Inc., Chicago, IL, USA), was used for data processing and analysis. Continuous variables were compared using the independent sample *t*-test, and categorical variables using the Pearson χ2 test (continuity corrected χ2 when minimum expected count was < 5; Fisher’s exact test was used when minimum expected count was < 1). The continuous variables were expressed as mean (T ± SD) or median. Corrected *P* < 0.05 was accepted as statistically significant. All reported *P* values were 2-sided.

## Results

### Case report

#### Patient 1

**Fig. 1:**
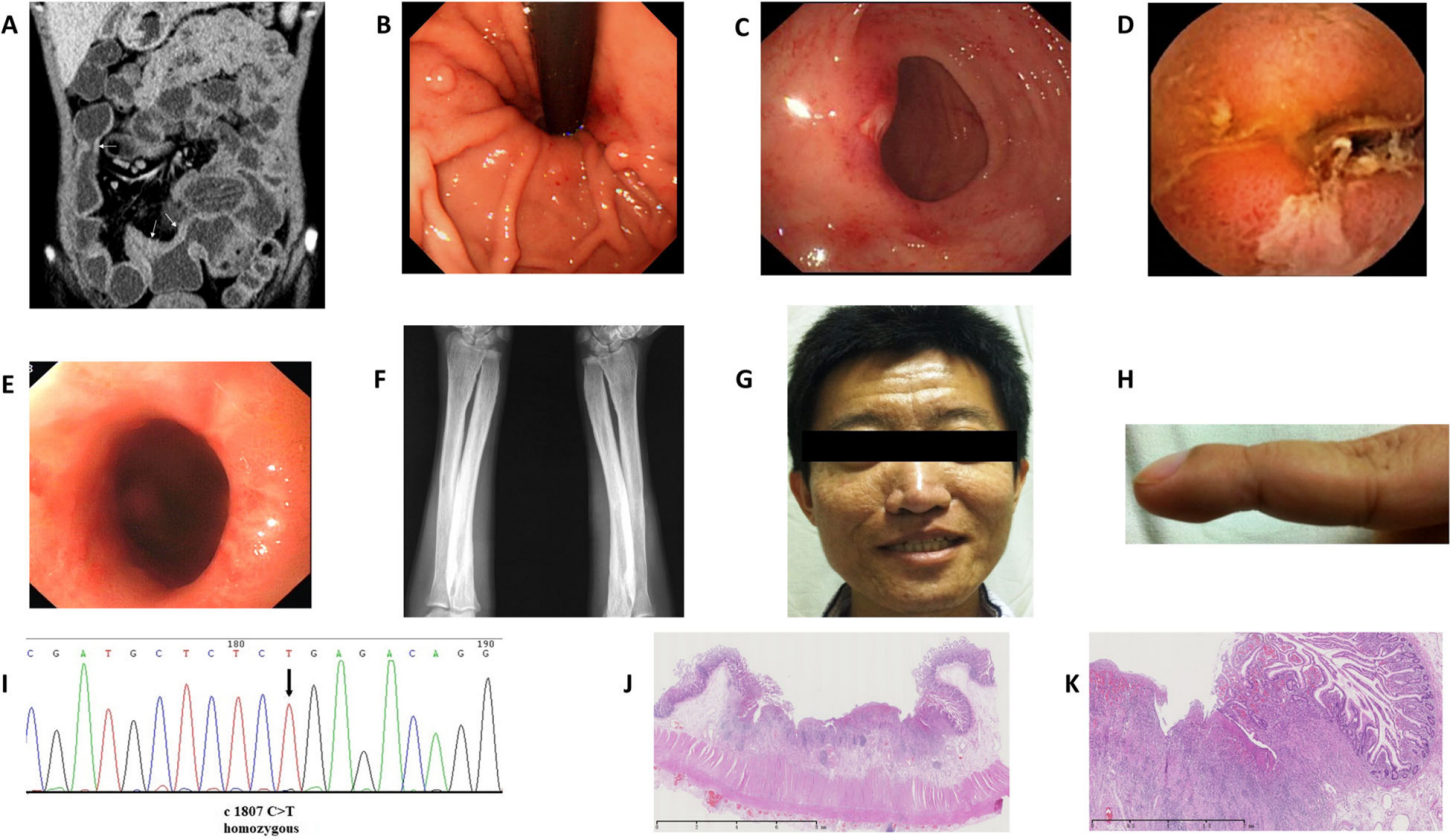
Images of patient 1. **a**. Contrast-enhanced CT showed abnormal enhancement of the mucosa and thickening of the small intestinal wall. **b**. Gastroscopy showed fundic gland polyps. **c**. the ulcer and hemorrhagic spots on the ileocecal valve. **d**. Ulcer of the ileum by capsule endoscopy. **e**. Ileum stenosis by double balloon enteroscopy. **f**. Periostosis of ulna and radius. **g**. Skin thickening and furrowing on the face. **h**. Clubbed finger. **i**. GeneScreen display of *SLCO2A1* mutation (homozygous c1807 C > T, R603X). **j**–**k**. HE stain of the ileum: Superficial ulcers involving mucosa and submucosa of the small intestine

A 28-year-old male was admitted on November 27, 2013. He complained of diarrhea (loose stool, three to five times per day) for over 10 years and hematochezia for about 1 month. The patient had been diagnosed with iron deficiency anemia 5 months after birth, and his hemoglobin level remained 70–80 g/L (normal range 110–150 g/L) for most of the time. On admission, his albumin level was 26 g/L (normal range 35–52 g/L). Results of liver and renal function were otherwise normal. Abdominal contrast-enhanced CT showed diffuse bowel wall thickening in jejunum and ileum, with abnormal enhancement of the small intestine mucosa (Fig. 1a). Gastroscopy showed chronic superficial gastritis and fundic gland polyps (Fig. 1b), and the Helicobacter pylori rapid urease test (Hp-RUT) was negative. Colonoscopy found scattered ulcers and hemorrhagic spots at the terminal ileum and colon (Fig. 1c). Capsule endoscopy and double balloon enteroscopy revealed multiple ulcers and stenosis of the ileum (Fig. 1d and e). Biopsy revealed unspecific gland hyperplasia and interstitial edema. ^99^Tc^m^-HAS (Human Serum Albumin) imaging confirmed protein leakage in the small intestine.

His diarrhea and hematochezia persisted despite experimental treatment of mesalazine and probiotics. The intestinal lesion (ulceration, hemorrhage and luminal stenosis) progressed, and anemia and hypoalbuminemia became refractory. Multiple periostosis was found in the extremities by X-ray (Fig. 1f). The diagnosis of PHO was then considered. In retrospect, the patient reported progressive thickening and furrowing of the skin on his face and enlargement of his fingertips since several years ago (Fig. 1g and h). He also admitted recurrent arthralgia in the knee and ankle joints. A bone marrow biopsy showed myelofibrosis. The genetic test confirmed *SLCO2A1* mutation with homozygous c1807 C > T, R603X (Fig. 1). He was diagnosed with PHO based on clinical characteristics, radiological findings, and gene mutation.

After treated with etoricoxib 30 mg~ 60 mg once daily (a type of cyclooxygenase-2 (COX-2) inhibitor [7–9]), arthralgia and the skin lesions were improved. Hematochezia and edema, however, persisted despite the use of nonsteroidal antiinflammatory drugs (NSAIDs) and supportive care followed by incomplete intestinal obstruction. Partial enterectomy was performed in December 2015 and January 2018, separately. Histopathological examination of the resected intestine showed chronic bowel inflammation with multifocal superficial ulcers involving mucosa and submucosa of the small intestine, and fibrogenic response in submucosa under the ulcers. The blood vessels in the intestinal wall were dilated (Fig. 1j and k). The muscularis propria and serosa were normal as well as mucosa between the ulcers. Exclusive enteral nutrition was administered after the second surgery, and his diarrhea, anemia, and hypoalbuminemia were improved.

#### Patient 2

**Fig. 2:**
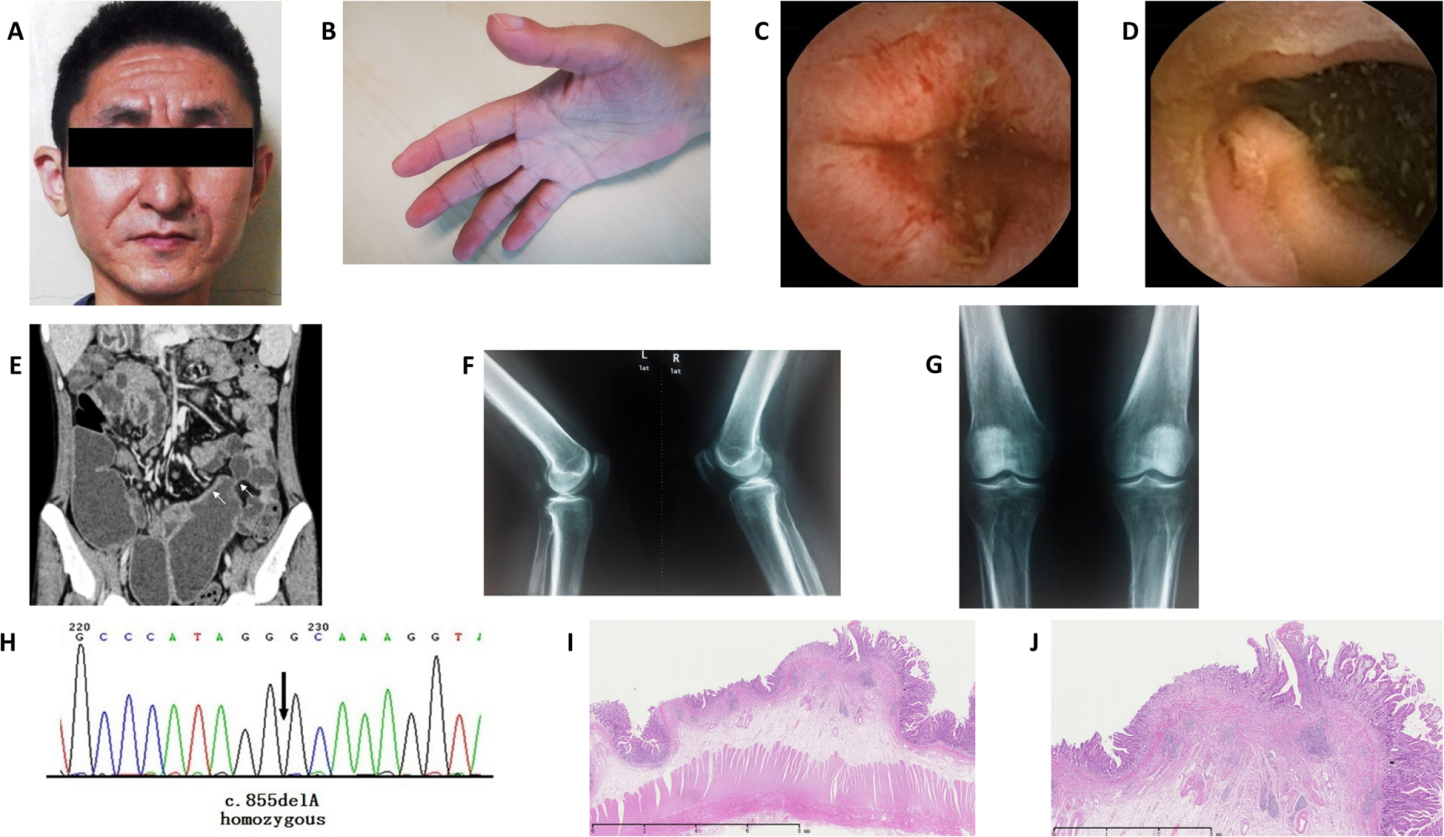
Images of patient 2. **a**. Pachydermia of the face. **b**. Clubbed Figs. **c**–**d**. Multiple ulcers in the ileum by capsule endoscopy. **e**. Abdominal contrast-enhanced CT revealed ileum wall enhancement, stenosis and dilated intestinal lumen. **f**–**g**. Periostosis of distal ulna and radius, distal femur and proximal tibia and fibula. **h**. GeneScreen display of *SLCO2A1* mutation (homozygous c.855delA, A286QfsX35). **i**–**j**. HE stain of the ileum: Chronic inflammation of small intestinal mucosa, with desmoplasia in the submucosal layer

A 36-year-old man was admitted on November 20, 2014, with typical pachydermia and digital clubbing (Fig. 2a and b). The patient had suffered intermittent abdominal colic, diarrhea, and anemia for 14 years before. He also reported arthralgia in both knees. The patient’s symptoms remained unexplained until 2012 when a dermatologist noticed that his facial skin was thickened and furrowed. The patient was diagnosed with PHO then, and his skin and joints symptoms were alleviated on the treatment of etoricoxib 60 mg once daily.

His diarrhea and arthralgia aggravated at the beginning of 2014, and his hemoglobin was lower than 60 g/L. He also gained edema and was diagnosed with hypoalbuminemia (albumin 22–27 g/L). Gastroscopy showed chronic superficial gastritis, and the Hp-RUT was negative. Capsule endoscopy discovered multiple ulcers in the small intestine with extensive mucosal erosion surrounding the ulcers (Fig. 2c and d). On admission, high C-reactive protein (CRP) level was detected. The fecal immunochemical test was positive. Abdominal contrast-enhanced CT revealed segmental dilated ileum with enhancement of intestinal wall (Fig. 2e). Radiography showed irregularly thickened cortex of distal ulna and radius, distal femur and proximal tibia and fibula (Fig. 2f and g). The magnetic resonance imaging of knees also showed periostosis of the articular surface. The patient and his two sisters were all found to have *SLCO2A1* gene mutation with homozygous c.855delA, A286QfsX35 (Fig. 2h), though his sisters had no symptoms related to PHO. After the treatment of mesalazine (3 g/day) for 3 months and prednisone (0.8 g/kg/day) for 1 month, anemia and hypoalbuminemia persisted, and incomplete intestinal obstruction worsened. Partial enterectomy was performed to relieve intestinal stenosis, and the postoperative pathological inspection showed chronic inflammation of small intestinal mucosa, with multifocal erosions and superficial ulcerations located in the mucosal layer, with desmoplasia in the submucosal layer (Fig. 2j and j). Diarrhea, anemia, and hypoalbuminemia were improved after the surgery.

### Literature search

We included 158 Chinese patients from 79 case reports written in Chinese (as reported in Additional file 1.) and 12 articles published in English [7–17] within a time range from January 2000 to April 2018.

### Clinical manifestations

**Fig. 3:**
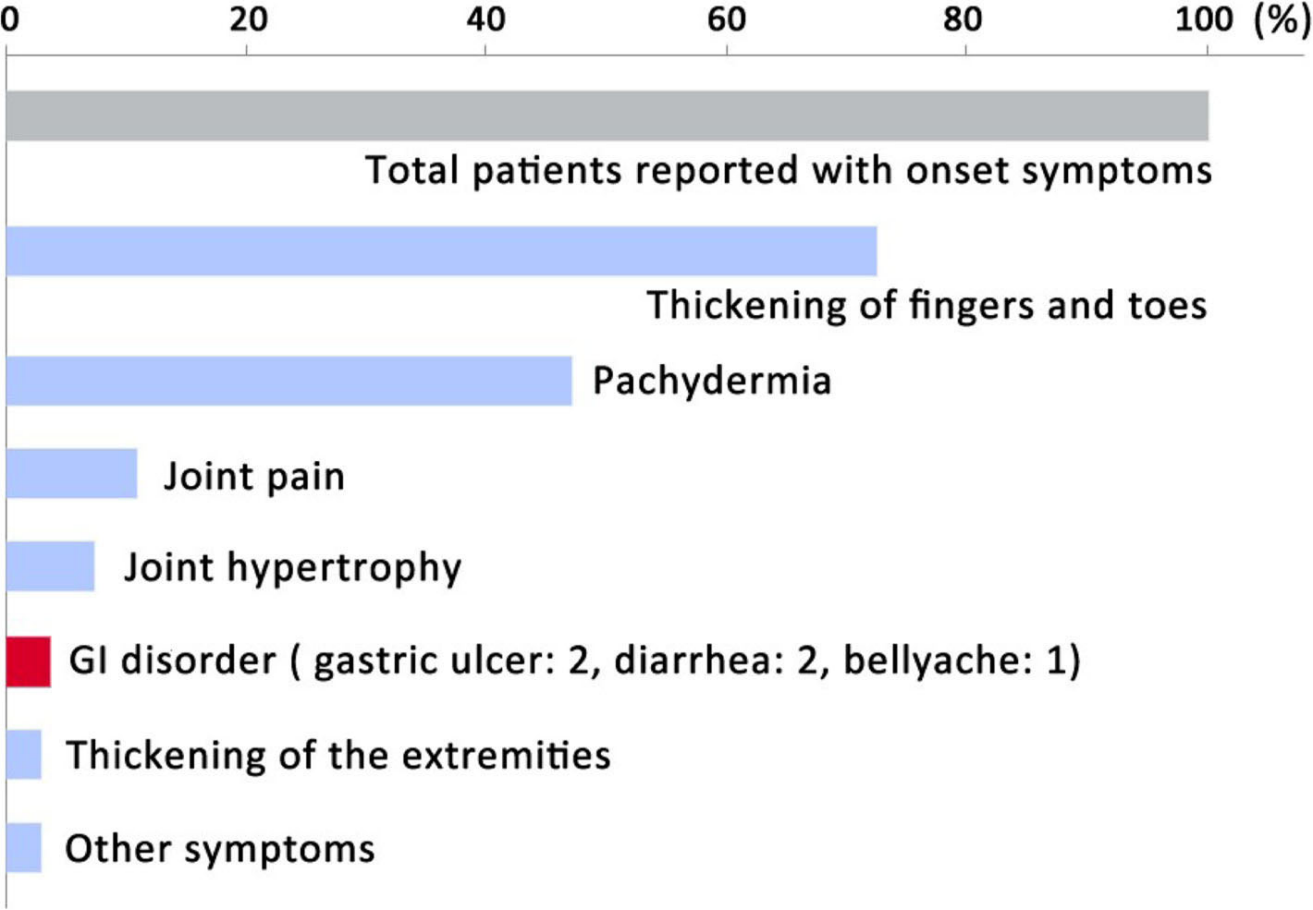
Onset symptoms of PHO patients

Among the 158 patients, 149 are male, and 9 are female. The age of disease onset was reported in 148 patients with a median age of 14 (range from 0 to 39) years old. The onset symptoms were reported in 138 patients (Fig. 3). Digital clubbing was the most common initial symptom (72.5%, 100/138). Pachydermia, or skin thickening of the face and head, was also common (47.1%, 65/138). Other onset symptoms include joint pain (10.9%, 15/138) and joint hypertrophy (7.2%, 10/138). Only a minority of patients (3.6%, 5/138) had GI disorders as their reporting symptom.

**Fig. 4:**
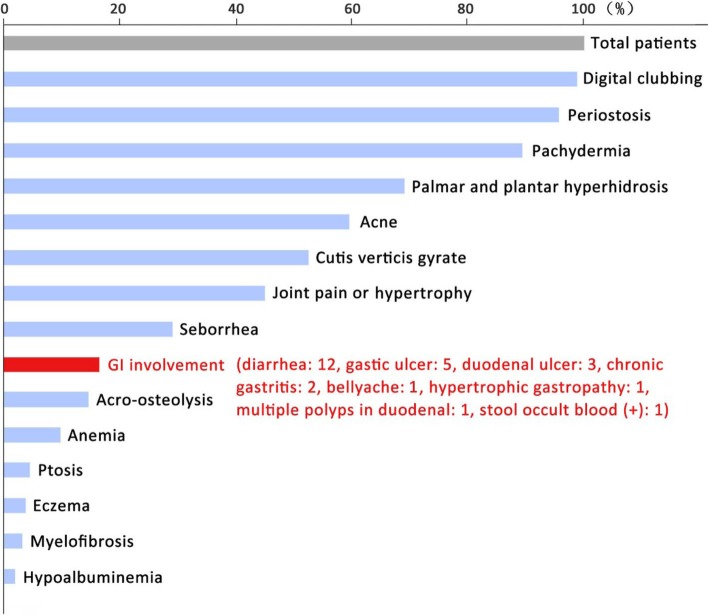
Symptoms and complications of PHO patients over the course

Throughout the disease, the patients exhibited various presentations (Fig. 4). Almost all patients developed digital clubbing (98.7%, 156/158) and periostosis (95.6%, 151/158). Acro-osteolysis (15.1%, 22/146) and myelofibrosis (3.8%, 5/133) were less common. Skin lesions included pachydermia (89.2%, 141/158), plantar hyperhidrosis (69.0%, 109/158), acne (59.5%, 94/158), cutis verticis gyrate (52.5%, 83/158), palmar and seborrhea (29.1%, 46/158), and eczema (3.8%, 6/158). Nearly half of the patients complained about joint pain or hypertrophy (44.9%, 71/158). 8 (5.1%) patients were reported ptosis due to eyelid thickening and enlargement. GI involvement over the course was 17.2% (26/151). Anemia (10.9%, 15/137) and hypoalbuminemia (3.7%, 5/136) were relatively rare. CRP was elevated in more than half of PHO patients (67.9%, 55/81).

### Genetics

In 49 patients with genetic test results, 13 had *HPGD* gene mutation (PHOAR1), and 36 had *SLCO2A1* gene mutation (PHOAR2). It is worth noting that most patients with GI involvement (86.7%, 13 /15) had *SLCO2A1* mutation.

### Gastrointestinal involvement

We summarized gastrointestinal manifestations in Table 1. A total of 26 cases (17.2%) had gastrointestinal involvement, including diarrhea (46.2%, 12/26), gastric ulcer (19.2%, 5/26), duodenal ulcer (11.5%, 3/26), chronic gastritis (7.7%, 2/26), bellyache (3.8%, 1/26), hypertrophic gastropathy (3.8%, 1/26), duodenal polyps (3.8%, 1/26) and occult GI bleeding (3.8%, 1/26). Only 5 patients had GI complaints at the beginning of the disease. Besides the two patients introduced above, one of the additional three people suffered from diarrhea for more than 10 years before diagnosis, and the other two had gastric ulcers. We compared the clinical characteristics of PHO patients with gastrointestinal complications against those without (Table 1). There was no statistically significant difference of the skin changes and bone abnormalities between the two groups. However, the incidences of anemia, hypoalbuminemia, and myelofibrosis *were more frequent in the* PHO patients with gastrointestinal involvement than those without (*P* < 0.01). In all of the PHO patients with gastrointestinal involvement, only two cases (reported above) had the surgical histopathological investigation, which showed multifocal superficial ulcers within mucosa and submucosa layer of the small intestine.

**Table 1:** Clinical and genetic data of 151 Chinese patients with primary hypertrophic osteoarthropathy

	Patients without GI involvement (*N* = 125)	Patients with GI involvement (*N* = 26)	*P* value
Age of onset	14.2 ± 4.2	15.4 ± 5.3	0.293
Age of diagnosis	23.7 ± 8.	25.9 ± 5.	0.158
Sex (Male)	92.8%(116/125)	100%(26/26)	0.244
Subtype			0.419
1	73.6%(92/125)	84.6%(22/26)	
2	23.2%(29/125)	11.5%(3/26)	
3	3.2%(4/125)	3.8%(1/26)	
Family history	16.8%(21/125)	23.1%(6/26)	0.588
Pachydermia	88.0%(110/125)	96.2%(25/26)	0.507
Cutis verticis gyrate	52.9%(63/119)	60.0%(15/25)	0.444
Eczema	3.2%(4/124)	4.0%(1/25)	0.543
Seborrhea	57.3%(71/124)	73.1%(19/26)	0.157
Acne	25.0%(29/116)	48.0%(12/25)	0.022
Hidrosis	70.2%(87/124)	61.5%(16/26)	0.861
Ptosis	5.5%(6/109)	9.5%(2/21)	0.621
Digital clubbing	99.2%(124/125)	96.2%(25/26)	0.375
Joint hypertrophy	95.1%(116/122)	92.3%(24/26)	0.679
Joint pain	40.0%(50/125)	50.0%(13/26)	0.347
Periostosis	96%(120/125)	96.2%(25/26)	1.000
Osteolysis	14.2%(17/120)	19.2%(5/26)	0.547
Bone age delay	1.0%(1/103)	0(0/20)	1.000
Cranial suture widening/delayed closure of the fontanelle	3.1%(3/96)	4.5%(1/22)	0.567
Hypoevolutism	0.8%(1/125)	0(0/26)	1.000
Anemia	4.5%(5/112)	40%(10/25)	< 0.001
Hypoalbuminemia	0.9%(1/112)	16.7%(4/24)	0.003
*Myelofibrosis*	0.9%(1/112)	19.0%(4/21)	0.002
Mutation of *SLCO2A1*	67.6%(23/34)	86.7%(13/15)	0.293

## Discussion

In this study, we reported two PHO patients with gastrointestinal complications in detail, and we summarized the clinical and genetic features of PHO in Chinese population. In China, although GI involvement in PHO patients had ever been mentioned in some studies [6, 18, 19], the clinical features of GI disorders were still unclear. This study systematically introduces clinical, endoscopic, and pathological characters of GI involvement in Chinese PHO patients.

PHO is a rare genetic disease with indistinct etiology and various complications. The diagnostic criterion of this disease is based on clinical characteristics including pachydermia, digital clubbing and periostosis [2]. Three clinical subtypes have been proposed: (1)a complete form, presenting the full-blown phenotype, (2) an incomplete form, with isolated bone involvement and limited skin changes and (3) a fruste form, with pachydermia and minimal or absent periostosis [2]. Diagnosis is often difficult when the onset of symptoms is incomplete or atypical. Gastrointestinal abnormalities are additional features of PHO with a reported incidence of 10.4%~ 12.2% [2] and can be neglected easily. Here we reported the incidence of gastrointestinal complications in Chinese PHO patients was 17.2%, unrelated to the three clinical subtypes. Both *SLCO2A1* and *HPGD* gene mutations can inactivate prostaglandin transport and degradation, resulting in persistent elevated serum PGE2 levels, which are likely to cause the clinical features of PHO [3, 4]. As elevated levels of PGE2 in gastrointestinal tissues are commonly known to protect against mucosal inflammation via the prostaglandin receptor EP3/EP4 [20, 21], the pathogenesis of gastrointestinal involvement in PHO patients needs to be clarified in future studies.

Though GI disorder was a rare onset symptom of PHO, with the progression of the disease, 17.2% (26/151) of the patients eventually developed this complication. Patients who had digestive tract involvement mainly suffered from diarrhea, gastric or duodenal ulcer and chronic gastritis. In our study, among the 15 patients with GI involvement who underwent genetic tests, 13 patients (86.7%) had *SLCO2A1* mutations (PHOAR2). As to another 2 patients who had *HPGD* mutations (PHOAR1), the GI disorders only manifested diarrhea without anaemia or hypoalbuminemia. In a study including 43 Chinese patients, watery diarrhea occurred in more than half of the patients with either *HPGD* or *SLCO2A1* gene mutation, but *SLCO2A1* mutated patients had a higher frequency of GI hemorrhage [19]. Similar findings were also reported by Hou et al. that diarrhea occurred in both *HPGD* and *SLCO2A1* defective patients but peptic ulcer and chronic gastritis only affected patients with a defective *SLCO2A1* gene [18]. Besides, Umeno et al. [5, 22] reported a rare autosomal recessive inherited enteropathy related to *SLCO2A1* gene mutation (CEAS), which could present intestinal abnormalities in isolation such as abdominal pain, diarrhea, bowel obstruction, ulcer, and hemorrhage. All these results may imply that GI involvement in PHO patients is more closely related to *SLCO2A1* rather than *HPGD* gene mutation. As only 2 PHOAR1 patients with GI involvement were included in our study, we couldn’t make further meaningful comparisons between subgroups of PHOAR1 and PHOAR2. More detailed data about *HPGD* gene mutation PHO patients with GI involvement need to be collected, and the underlying mechanism for the preference of mutation in *SLCO2A1* in PHO patients with GI involvement awaits further studies.

In this study, PHO patients with GI involvement were more likely to have anaemia, hypoalbuminemia, and myelofibrosis. Gastrointestinal ulceration can lead to bleeding and albumin loss, then cause anaemia and hypoalbuminemia. Zhang Z. et al. also reported anaemia and hypoalbuminemia in PHO patients with watery diarrhea [6]. Some studies suggested that PHO patients with prostaglandin transporter *SLCO2A1* mutations were more likely to develop myelofibrosis [2, 14], which might explain the high incidence of myelofibrosis in PHO patients with GI involvement. *SLCO2A1* mutation with homozygous c1807 C > T, R603X was confirmed in the reported patient 1, and compound heterozygous mutations of this site had ever been described in both PHO and CEAS patients [22, 23]. The *SLCO2A1* gene mutation with homozygous c.855delA, A286QfsX35 in our patient 2 had also been described by Zhang et al. in PHO patients [6].

GI lesions in PHO have unique morphological characteristics. Similar to the clinical features reported in the study of Umeno et al. [5, 22], the two patients in our study also had the lesions of multiple ulcers varied in shape with or without luminal stenosis, and persistent gastrointestinal bleeding and protein losing. We also reported the unique histologic changes in PHO patients with GI lesions. The erosions, ulcerations and fibroblastic proliferation were restricted within the mucosa and submucosa layer, which were different from Crohn’s disease but similar to cryptogenic multifocal ulcerous stenosing enteritis (CMUSE). CMUSE is a rare condition affecting the small intestine first described by Debray et al. in 1964 [24]. Typical clinical picture of CMUSE includes skipping ulceration and stenosis without systematic inflammatory response [25]. The etiology and pathogenesis of CMUSE are largely unknown. However, CMUSE was shown to be an autosomal recessive inherited disease caused by mutations in the *PLA2G4A* gene [26], and patients with CMUSE generally have normal CRP levels and respond well to steroid therapy. The two patients we reported here had elevated CRP levels and failed to respond to prednisone treatment. Hence, we postulate that CMUSE and HPO involving GI tract might be two different entities.

Recently Umeno et al. suggested that chronic enteropathy associated with *SLCO2A1* gene mutation (CEAS), also known as chronic nonspecific multiple ulcers of the small intestine with SLCO2A1 mutation (CNSU), was a new clinical entity, distinct from Crohn’s disease and other known inflammatory bowel disorders such as intestinal Behcet’s disease and NSAIDs-induced enteropathy. Some of the CEAS patients in their study had PHO characters based on digital clubbing, periostosis, and pachydermia, and 5 male patients fulfilled the major clinical criteria of PHO [5]. The two cases reported in our study also fit the characters of CEAS, which is defined as an entity characterized by multiple small intestinal ulcers caused by SLCO2A1 mutations with nonspecific histology and chronic persistent gastrointestinal bleeding. The relationship between CEAS and PHO merits debate. No doubt they have overlapping clinical features. In this systematic review, however, all of the PHO patients with GI involvement were males. CEAS was more common in females with a reported gender preference of 71.7%~ 77.8% [5, 22]. Interestingly, all of the CEAS patients who also met the diagnostic criteria of PHO were males, and no female CEAS patient developed PHO [5, 22]. Based on these findings we postulate that the SLCO2A1 gene mutation might be a trigger factor for both PHO and CEAS, but other factors including gender should modify the progress of the two diseases.

There is no consensus for the treatment of gastrointestinal involvement in PHO till now. Unlike inflammatory bowel disease, mesalazine and prednisone were ineffective in our patients. Although surgical resection of the diseased intestine can temporarily relieve symptoms, specific medical treatment is pressingly required nevertheless. Exclusive enteral nutrition (EEN) has been tried with relative success in one of the two patients, but the long-term effectiveness of EEN awaits validation. SLCO2A1 mutations inactivate prostaglandin E2 (PGE2) transporter and cause excessive release of PGE2, leading to symptoms in PHO. For instance, Zhang, et al. reported that the urinary PEG2 levels of SLCO2A1 mutation PHO patients were significantly higher than healthy controls [6]. NSAIDs can improve skin and bone lesions of PHO patients by inhibiting the production of PGE2, which is effective both in PHOAR1 and PHOAR2 patients [19, 27]. Otherwise, no study had ever reported the effect of NSAIDs for alleviating GI involvement in PHO patients. NSAIDs failed to ameliorate GI lesions in the two patients reported in this study, although their skin and joints symptoms were improved. A possible explanation is that the elevated levels of PGE2 in gastrointestinal tissues are known to protect against mucosal inflammation via the prostaglandin receptor [28]. As a matter of fact, the adverse events of NSAIDs-related gastrointestinal injury are just due to the ability of these agents to suppress prostaglandin synthesis. Therefore, it is not unlikely that the use of NSAIDs to treat PHO patients with GI involvement tend to be ineffective, especially in PHOAR2 patients. In addition, it was reported that NSAIDs are associated with excessive production of vasodilating molecules such as inducible nitric oxide [29]. The remarkable dilated microvessels on the histological investigation in this study imply that vasodilation is at least a contributing factor in GI lesions of PHO, which in turn aggravates mucosal inflammation and chronic hemorrhage. Further studies are eagerly awaited to determine the medical treatment, other than NSAIDs, for GI complications in PHO.

## Conclusions

The gastrointestinal complication is uncommon and unique in patients with PHO, leading to intestinal ulceration and stenosis. PHO patients with GI involvement are more likely to have anemia, hypoalbuminemia, and myelofibrosis. Mutations of *SLCO2A1* might be the pathogenic trigger. Conventional treatment of NSAIDs may not be effective for PHO patients with gastrointestinal complications.

## Supplementary information

**Additional file 1.** 79 case reports written in Chinese.

## Data Availability

The datasets generated during and/or analyzed during the current study are available on PUBMED, EMBASE, Cochrane Library and the China National Knowledge Infrastructure (www.cnki.net) database from January 1, 2000 to April 30, 2018, as reported on references and Additional file 1.

## Abbreviations

CEAS: chronic enteropathy associated with *SLCO2A1* gene
CMUSE: cryptogenic multifocal ulcerous stenosing enteritis
COX-2: cyclooxygenase-2
CRP: C-reactive protein
EEN: Exclusive enteral nutrition
GI: gastrointestinal
HO: Hypertrophic osteoarthropathy
PGE2: prostaglandin E2
PHO: Primary hypertrophic osteoarthropathy
SPSS: The Statistical Package for Social Sciences
