# Supplementary material for: Primary hypertrophic osteoarthropathy related gastrointestinal complication has distinctive clinical and pathological characteristics: two cases report and review of the literature
Source: Orphanet J Rare Dis. 2019 Dec 26;14:297. doi: 10.1186/s13023-019-1264-5 (PMC6933916; doi:10.1186/s13023-019-1264-5)
Supplement: Supplementary file 1 — Additional file 1. 79 case reports written in Chinese. [file 13023_2019_1264_MOESM1_ESM.docx]

.

1. Luo B, Guo Z, Zhang J. One case: pachydermoperiostosis. Journal of Practical Radiology. 2000; 16:573.
2. Wang C, Cai F. Three cases of familial pachydermoperiostosis in a family. Chinese Journal of Medical Genetics. 2000; 17: 86.
3. Zou Q, Meng K. One case report of pachydermoperiostosis. Radiologic Practice. 2001; 16: 373.
4. Chen Y, Cai X. One case report of primary hypertrophic osteoarthropathy with literature analysis. Chinese Journal of Difficult and Complicated Cases. 2002; 1: 159-60.
5. Zhang G, Luo J, Li H, Chen L, Xu G. One case report of pachydermoperiostosis. Journal of Rare and Uncommon Diseases. 2002; 9: 56-7.
6. Zhang W, Zhang Q, Bao C. Primary hypertrophic osteoarthropathy. Shanghai Medical Journal. 2002; 25: 434-5.
7. Chen L. One case report of primary hypertrophic osteoarthropathy. Chinese Journal of Rheumatology. 2003; 7: 383-4.
8. Liu W, Liu L, Li S. Surgical treatment of facial pachydermoperiostosis. Chinese Journal of Medical Aesthetics and Cosmetology. 2003; 17: 41-2.
9. Chang C, Zhang W, Feng X. Two cases of pachydermoperiostosis. The Chinese Journal of Dermatovenereology. 2004; 18: 296-7.
10. Chen X, Qi N, Liu G, Zhao Z, Liu Z, Chen K. Primary hypertrophic osteoarthropathy (A report of 4 cases and review of the literature). Journal of Practical Radiology. 2005; 21: 54-7.
11. Hu Z, Jiang X, Zhu Y. Primary hypertrophic osteoarthropathy：2 cases report and a review of literature. Journal of Rare and Uncommon Diseases. 2005; 12: 13-5.
12. Gan G, Qu T, Fang K, Wang B. A case of pachydermoperiostosis. The Chinese Journal of Dermatovenereology. 2005; 19: 554-5.
13. Hou Y, Zhang X, Zhang Z, Zeng X, Zhang F, Yu M. Clinical analysis of Primary hypertrophic osteoarthropathy (report of 5 cases). Beijing Medical Journal. 2006; 28: 734-7.
14. Liu Z. A case of primary pachydermoperiostosis. Papers of the Fifth Academic Symposium on Dermatology of Integrated Traditional Chinese and Western Medicine in Jiangxi Province. 2006: 66-7.
15. Li D, Dai C, Han H, Qiu M. Two cases report of pachydermoperiostosis. Tianjin Medical Journal. 2006; 34: 357-8.
16. Li A, Qin H, Xu L, Yang Z, Wang Y. Pachydermoperiostosis: a report of 2 familial cases. Journal of Practical Radiology. 2006; 22: 534.
17. Liang Y, Yan X. A case of pachydermoperiostosis. Journal of the Fourth Military Medical University. 2006; 27: 1824.
18. Wang G, Han S, Yu M, Liu J, Yao Z, Li W. Primary hypertrophic osteoarthropathy: a case report and literature review. Beijing Medical Journal. 2006; 28: 412-4.
19. Zhu J, Zeng X. A case of pachydermoperiostosis. Papers Compilation of Beijing Annual Conference on Rheumatism in 2006. 2006: 177-8.
20. Yue X, Wu R, Wang A, Zhu X. A case report of pachydermoperiostosis. The Chinese Journal of Dermatovenereology. 2007; 21: 495-6.
21. Lin X, Ma L, Ai M, Wu D. A case of primary hypertrophic osteoarthropathy with literature review. Chinese Journal of Rheumatology. 2008; 12: 263-5.
22. Liu Y, Zhao B, Gong Y. A case of pachydermoperiostosis. Journal of Clinical Dermatology. 2008; 37: 118-9.
23. Man S. Hypertrophic osteoarthropathy. Papers Compilation of Beijing Annual Conference on Rheumatism in 2008. 2008: 20-3.
24. Mayi N, Yang Q, Yan L, Wu X. Pachydermoperiostosis misdiagnosed as acromegaly: a case report. Infection, Inflammation, Repair. 2008; 9: 220.
25. Yang X, Liu Y, Chen L. A case of pachydermoperiostosis. Chinese Journal of Aesthetic Medicine. 2008; 17: 1743.
26. Li Q, Tu Y, Wu S, Zhou J, Bao Y, Jia W, et al. Two cases report of pachydermoperiostosis. Journal of Shanghai Jiaotong University (Medical Science). 2008; 28: 614-5.
27. Deng A, Song D, Liu J. A case report of pachydermoperiostosis. Journal of Chinese Physician. 2009; 11: 1151.
28. Huang H, Deng J, Yang X, Zhong B. A case of pachydermoperiostosis. Journal of Clinical Dermatology. 2009; 38: 464-5.
29. Lou A, Guo W, Zhi F. Pachydermoperiostosis with giant duodenal ulcer: a case report.  Chinese Journal of Digestive Endoscopy. 2009; 26: 278-80.
30. Man S, Song H, Wu H. Clinical analysis of hypertrophic osteoarthropathy. Chinese Journal of Rheumatology. 2009; 13: 845-7.
31. Qiu M, Tao S, Gu J. A case of pachydermoperiostosis. Chinese Journal of Dermatovenereology of Integrated Traditional and Western Medicine. 2009; 8: 110-1.
32. Wen Z, Li J, Zhang L. A case of pachydermoperiostosis. Chinese Journal of Rheumatology. 2009; 13: 508-9.
33. Wang H, Zhao M. A case of primary hypertrophic osteoarthropathy. Xinjiang Medical Journal. 2009; 39: 108-10.
34. Zhou N, Wang K, Zhang Z. A case report of primary hypertrophic osteoarthropathy. Journal of Clinical Pediatrics. 2009; 27: 789-90.
35. Chen Z. A case report and literature review about primary hypertrophic osteoarthropathy. Medical Innovation of China. 2010; 7: 167-8.
36. Ning J, Liu X. A case of pachydermoperiostosis. Radiologic Practice. 2010; 25: 1182.
37. Jia H, Wen S. A case report of pachydermoperiostosis. Shandong Medical Journal. 2010; 50: 64.
38. Peng Z. A report of one primary pachydermoperiostosis case. Papers Compilation of the Congress of Cosmetologists and Plasticians of the Chinese Medical Association. 2010: 789-91.
39. Yang Y, Wang S, Gao T. A case of pachydermoperiostosis. Journal of Clinical Dermatology. 2010; 39: 372.
40. Zhang C, Wei G, Song H, Ma D, Zhang D. A case of pachydermoperiostosis. China Journal of Leprosy and Skin Diseases. 2010; 26: 281-2.
41. Chen X, Chen Y, Wu Z. Five cases analysis of pachydermoperiostosis. Journal of Rare and Uncommon Diseases. 2011; 18: 33-5.
42. Chen C, Wang X, Lai c, Dong G, Liang L, Fu J. Pachydermoperiostosis in a girl. Chinese Journal of Applied Clinical Pediatrics. 2011; 26: 910.
43. Du T, Zhao H, Liu L, Yu B. Surgical treatment of facial pachydermoperiostosis. Chinese Journal of Medical Aesthetics and Cosmetology. 2011; 17: 147-8.
44. Hu J, Gao L, Li Q, Wang G. A case of pachydermoperiostosis. Journal of Practical Dermatology. 2011; 04: 60-1.
45. Li Y, Wang K, Yan G. A case of primary hypertrophic osteoarthropathy. Chongqing Medicine. 2011; 40: 1248.
46. Liu L, Zhao L. A case report of pachydermoperiostosis. Contemporary Medicine. 2011; 17: 59-60.
47. Liu J, Zhao Y, Jiang D, Guo Z, Wang L, Yu M. Primary hypertrophic osteoarthropathy: 3 cases report and literature review. Chinese Journal of Clinicians (Electronic Edition). 2011; 05: 3738-41.
48. Yu S, Xiang F, Ju H, Pu X. A case of primary pachydermoperiostosis. Chinese Journal of Medical Genetics. 2011; 28: 479.
49. Zhang L, Dai Y, Liu D. A case of pachydermoperiostosis. The Chinese Journal of Dermatovenereology. 2011; 25: 73.
50. Bian Y, Weng X, Yang B, Zhai J, Sun T. Arthroscopic synovectomy for pachydermoperiostosis: a case report. Acta Academiae Medicinae Sinicae. 2012; 34: 651-5.
51. Duan J. A case of primary hypertrophic osteoarthropathy. Journal of Dermatology and Venereology. 2012; 34: 244.
52. Deng J, Zhong X, Liu G, He Z, Zhang J, Bai J, et al. A case of complete primary hypertrophic osteoarthropathy. Chinese Journal of Clinicians (Electronic Edition). 2012; 06: 211-2.
53. Fang J, Yuan W. A case of pachydermoperiostosis. The Chinese Journal of Dermatovenereology. 2012; 26: 1124-5.
54. Li T. Primary hypertrophic osteoarthropathy misdiagnosed as acromegaly: a case report. Shandong Medical Journal. 2012; 52: 102.
55. Li S. Clinical characteristics and treatment of pachydermoperiostosis. Guide of China Medicine. 2012; 31: 612-3.
56. Liu L, Li G, Chang H. A case of pachydermoperiostosis. International Journal of Dermatology and Venereology. 2012; 38: 423.
57. Liu S, Qiu J, Meng Y, Qiu Z. A Chinese girl with primary hypertrophic osteoarthropathy (PHO) caused by homozygous novel deletion in HPGD. Basic and Clinical Medicine. 2012; 32: 656-9.
58. Liu P, Sun J. A case of pachydermoperiostosis. The Chinese Journal of Dermatology. 2012; 45: 52.
59. Liu Y, Zeng W, Geng S, Yi Q. Pachydermoperiostosis complicated by pulmonary cyst and scoliosis: a case report and review of the literature. Journal of Clinical Dermatology. 2012; 41: 542-4.
60. Sun Z, Dai C, Qiu M. Four cases of pachydermoperiostosis. Journal of Clinical Dermatology. 2012; 41: 38-9.
61. Song D, Xue M, Ye L. Imaging and clinical diagnosis of the pachydermoperiostosis. Journal of Chinese Clinical Medicine Imaging. 2012; 23: 825-7.
62. Wang Y, Du H. Primary hypertrophic osteoarthropathy: one case report and literature review. Zhejiang Medical Journal. 2012; 34: 639-41.
63. Wang Y, Liu Y, Ma J, Li C, Zhi C. A case of primary hypertrophic osteoarthropathy.  Chinese Journal of General Practitioners. 2012; 11: 158-9.
64. Xiang P, Gao L, Leng Q. A case report of pachydermoperiostosis. Acta Academiae Medicinae Zunyi. 2012; 35: 340-1.
65. Zhang T, Che D. Two cases of pachydermoperiostosis. Diagnosis and Therapy Journal of Dermato-Venereology. 2012; 19: 171-2.
66. Zhang Y, Wang X, Wang H. A case of pachydermoperiostosis. The Chinese Journal of Dermatovenereology. 2012; 26: 1124-5.
67. Li L, Li F. A case of pachydermoperiostosis with literature review. Orhtopaedics Journal. 2013; 4: 211.
68. Li J, Lin A, Huo Y. A case of pachydermoperiostosis with literature review. Jiangxi Medical Journal. 2013; 48: 35-7.
69. Liao Z. A case of pachydermoperiostosis. Health Must-read Magazine. 2013; 12: 522.
70. Jiao B. A case of primary hypertrophic osteoarthropathy. China Journal of Leprosy and Skin Diseases. 2014; 8: 502-3.
71. Li D, Wang F, Ma X, He R, Gu J. One case of pachydermoperiostosis. Chinese Journal of Endocrinology and Metabolism. 2014; 30: 705-7.
72. Tian Y, Shen F. A case of pachydermoperiostosis. Chinese Journal of Medical Aesthetics and Cosmetology. 2014; 20: 150-1.
73. Wu C, Jin H. A case of pachydermoperiostosis with slow circumfluence of the lymph in lower limbs. China Journal of Leprosy and Skin Diseases. 2014; 12: 750-1.
74. Wang Z, Tan L. A case of pachydermoperiostosis. Chinese And Foreign Medical Research. 2014; 3: 163-4.
75. Wang L, Yu J, Li Y, Liu X, Zhang C. Genetic diagnosis of a Chinese Han family with primary hypertrophic osteoarthropathy. Chinese Journal of Medical Genetics. 2015; 32: 213-7.
76. Yang H, Han S. A case of pachydermoperiostosis with multiple system abnormalities. Journal of Clinical Dermatology. 2015; 10: 669.
77. Ding C, Ren L, Le H, Li J, Zhao T, Qin G. A case of pachydermoperiostosis caused by a deletion mutation in the HPGD gene. Chinese Journal of Dermatology. 2016; 49: 47-9.
78. Li Y, Wang Y, Wang S, Xu Y. A case report of primary pachydermoperiostosis. Journal of Practical Dermatology. 2016; 9: 72-3.
79. Jin P, Zhang Q, He H, Zhu W, Long X, Mo Z. Two cases of primary hypertrophic osteoarthropathy with SLCO2A1 gene mutations. Journal of Central South University (Medical Sciences). 2018; 43: 100-5.
